# Rel/NF-κB Transcription Factors Emerged at the Onset of Opisthokonts

**DOI:** 10.1093/gbe/evab289

**Published:** 2022-01-06

**Authors:** Michelle M Leger, Núria Ros-Rocher, Sebastián R Najle, Iñaki Ruiz-Trillo

**Affiliations:** 1 Institute of Evolutionary Biology (Consejo Superior de Investigaciones Científicas-Universitat Pompeu Fabra), Barcelona, Catalonia, Spain; 2 Department of Genetics, Microbiology and Statistics, Institute for Research on Biodiversity, University of Barcelona, Catalonia, Spain; 3 Catalan Institution for Research and Advanced Studies (ICREA), Barcelona, Catalonia, Spain

**Keywords:** NF-κB, Rel homology domain, transcription factors, opisthokonts, NF-kappa B

## Abstract

The Rel/NF-κB transcription factor family has myriad roles in immunity, development, and differentiation in animals, and was considered a key innovation for animal multicellularity. Rel homology domain-containing proteins were previously hypothesized to have originated in a last common ancestor of animals and some of their closest unicellular relatives. However, key taxa were missing from previous analyses, necessitating a systematic investigation into the distribution and evolution of these proteins. Here, we address this knowledge gap by surveying taxonomically broad data from eukaryotes, with a special emphasis on lineages closely related to animals. We report an earlier origin for Rel/NF-κB proteins than previously described, in the last common ancestor of animals and fungi, and show that even in the sister group to fungi, these proteins contain elements that in animals are necessary for the subcellular regulation of Rel/NF-κB.

Significance

The Rel/NF-κB transcription factor family plays a fundamental role in animal innate immunity, programmed cell death, intercellular signaling, and transcriptional regulation. Here, we show that proteins sharing the characteristic features of animal Rel/NF-κB transcription factors originate much earlier than previously described: not in a relatively recent common ancestor of animals and some of their unicellular relatives, but prior to the divergence of animals and fungi. Intriguingly, we show that even the earliest-diverging nonmetazoan Rel/NF-κB-like protein has sequence features consistent with an animal-like mode of regulation.

The Rel homology region (RHR) is an evolutionarily conserved N-terminal DNA-binding region present in two major paralogous families of animal transcription factors with crucial roles in immunity and development: the Rel/Nuclear Factor-κB (NF-κB) and the Nuclear Factor of Activated T-cells (NFAT) families. Members of the Rel/NF-κB family were originally described in the late 1980s as oncogenes (Gilmore and Temin 1986) and immunoglobulin κ light chain enhancer-binding proteins in vertebrates (Sen and Baltimore 1986). Members of this family have since been implicated in a wide range of cellular processes in animals (Ghosh and Hayden 2012), including innate and adaptive immunity (Hayden and Ghosh 2011), cell cycle regulation (Ledoux and Perkins 2014), apoptosis (Kucharczak et al. 2003), autophagy (Salminen et al. 2012), and regulation of oxidative stress responses (Lingappan 2018). Despite these crucial roles in animals, their functions and domain architectures in other taxa remain to be fully explored. Pinpointing the origins and taxonomic distribution of Rel/NF-κB proteins is a fundamental first step to tackle these questions and understand the evolutionary history of Rel/NF-κB proteins. Rel/NF-κB proteins are characterized by the presence of an RHR, consisting of an N-terminal DNA-binding and dimerization domain that facilitates homo- or heterodimerization, and a short stretch of arginine and/or lysine residues forming a nuclear localization signal (NLS) that mediates the translocation of these proteins into the nucleus (fig. 1*A*) (reviewed in Napetschnig and Wu [2013] and Williams and Gilmore [2020]).

**Fig. 1.**
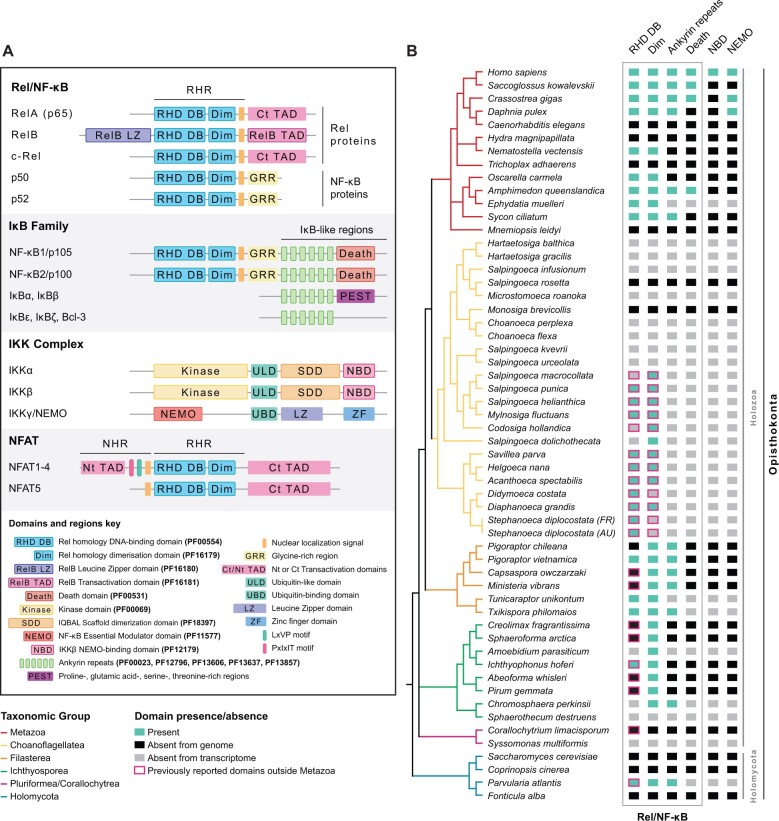
Rel/NF-κB proteins emerged at the onset of Opisthokonta. (*A*) Domain architecture representation of members of the Rel/NF-κB, and NFAT protein families, IκB, and the IKK complex. Details of the represented features are shown in the domains and regions key. The Rel Homology Region, characteristic of Rel/NF-κB and NFAT proteins, contains conserved Rel homology DNA-binding (RHD DB) and dimerization domains (Dim) and, in the case of Rel/NF-κB proteins, an NLS (orange bar). Rel proteins also contain a C-terminal, serine-rich Transactivation Domain (Ct TAD) or a RelB TAD; and RelB proteins additionally possess an N-terminal leucine zipper domain (RelB LZ). The NF-κB1 precursor (p105) and NF-κB2 precursor (p100) contain a more centrally located GRR and C-terminal Death domain (Death). These precursors share with IκB proteins C-terminal ankyrin repeats (light green bars). Other domains present in IκB families include proline-, glutamic acid-, serine-, and threonine-rich regions (PEST). Key domains specific to NFAT proteins, include an N-terminal TAD (Nt TAD) inside an NHR, an NLS (orange bar) and C-terminal TAD (Ct TAD). PxIxIT and LxVP Calcineurin-binding motifs in the NHR are depicted with a magenta and a green bar, respectively. Domains for IKKalpha and beta include kinase domains (Kinase); ubiquitin-like domains (ULD); ubiquitin-binding domains (UBD); IQBAL scaffold dimerization domain (SDD); NEMO-binding domains (NBD). Domains for IKKγ/NF-κB essential modulator (NEMO) include NEMO domain (NEMO); UBD; leucine-zipper domains (LZ); and zinc finger domains (ZF). (*B*) Presence or absence of key Pfam domains analyzed in this study are represented in columns and color-coded according to genome or transcriptome data source (indicated in the Domain presence/absence key). The phylogenetic relationships of selected taxa are based on several recent phylogenomic studies (Torruella et al. 2015; Grau-Bové et al. 2017; Hehenberger et al. 2017; Tikhonenkov et al. 2020; Urrutia et al. 2021). Taxa are color-coded according to the Taxonomic Group key.

The Rel/NF-κB family can be further subdivided into two classes according to the transactivation potential of its members. The first, collectively termed Rel proteins, include the vertebrate RelA (p65), RelB, and c-Rel and their orthologs (fig. 1*A*). Besides the RHR, Rel proteins also contain a poorly conserved C-terminal transactivation domain (TAD) that is acidic, and may be rich in proline, serine, glutamine, and/or hydrophobic residues (Bull et al. 1990; Blair et al. 1994; Gross et al. 1999), that allows them to activate target gene expression. RelB proteins additionally possess an N-terminal leucine zipper domain critical for their activity (Dobrzanski et al. 1993). Rel proteins can homodimerize, or heterodimerize with other Rel/NF-κB family members lacking a classical TAD (reviewed in Napetschnig and Wu [2013]).

The second class includes the vertebrate p50 and p52 and their orthologs (fig. 1*A*). p50 and p52 are synthetized as larger precursors termed NF-κB1/p105 and NF-κB2/p100, respectively. These precursors include an RHR followed by a glycine-rich region (GRR) and a variable number of C-terminal ankyrin repeats. In their inactive state, ankyrin repeats inhibit nuclear localization and transcriptional activity and keep the NF-κB dimers sequestered in the cytosol. They additionally possess a death domain that mediates interaction with other death domain-containing signaling proteins (Hayden and Ghosh 2008). The p105 and p100 precursors are classified as inhibitors of NF-κB (IκBs). This category also includes a family of separate C-terminal ankyrin repeat-containing proteins that carry out the same inhibitory function for Rel proteins, by sequestering them in the cytosol (Kanarek et al. 2010) (fig. 1*A*). Upon upstream activation, C-terminal serine residues in ankyrin-repeat-rich regions of IκBs are phosphorylated by an IκB kinase complex (IKK) (Karin 1999). The IKK complex is also involved in the processing of the p105 and p100 precursors, leading to the ubiquitination and proteasomal degradation of their C-terminal regions (fig. 1*A*). In this case, the proteasome falls off at the GRR located between the RHR and the C-terminal ankyrin repeats (Lin and Ghosh 1996; Williams and Gilmore 2020). This process releases an intact N-terminal part of the NF-κB protein, including the GRR (Moorthy et al. 2006), and leads to its nuclear translocation for gene expression activation (reviewed in Napetschnig and Wu [2013]). A key scaffolding component of the IKK complex, the IKKγ/NF-κB essential modulator (NEMO), is also required for IKK recruitment and NF-κB activation (fig. 1*A*) (reviewed in Napetschnig and Wu [2013]).

The NFAT family constitutes the paralogous group of RHR-containing proteins. NFAT proteins were first described almost three decades ago as calcium-dependent transcription factors implicated in T-cell activation (Shaw et al. 1988), cell proliferation, migration, and angiogenesis (reviewed in Müller and Rao [2010]). NFAT proteins contain a more centrally located RHR flanked by longer N- and C-terminal regions, and lack ankyrin repeats (fig. 1*A*). The NFAT NLS is contained not within the RHR, but within the N-terminal regulatory region known as the NFAT homology region (NHR) (fig. 1*A*). This region also contains calcineurin-binding sites required for nuclear translocation (Park et al. 2000). NFAT1-4 also possess N-terminal TADs (Serfling et al. 2004). NFAT5, the only noncalcium regulated NFAT protein in humans, lacks an NHR (fig. 1*A*) but is generally located in the nucleus, and plays a role in osmotic stress response and immune cell development (Lee et al. 2019).

Animals and their closest unicellular relatives together comprise the eukaryotic group Holozoa; the larger eukaryotic group comprising Holozoa, fungi, and their closest unicellular relatives, is known as Opisthokonta (fig. 1*B*). Rel homology domain-containing proteins were initially believed to be specific to animals (Metazoa), in which these proteins have been extensively studied. However, they were later reported in two lineages of unicellular holozoans: the filastereans (Mikhailov et al. 2009; Sebé-Pedrós et al. 2011) and choanoflagellates (Richter et al. 2018; Williams and Gilmore 2020). These sequences branched sister to all metazoan NF-κB and NFAT sequences, but contained RHRs, GRR, NLS, and/or ankyrin repeats more characteristic of the Rel/NF-κB family (Sebé-Pedrós et al. 2011; Williams and Gilmore 2020). Rel homology DNA-binding domains had also been reported as being present in more distantly related taxa, including a nucleariid and possibly one or more ichthyosporeans and/or pluriformeans ([de Mendoza et al. 2013; de Mendoza and Sebé-Pedrós 2019]; fig. 1*B* and supplementary table S1, Supplementary Material online). However, these studies focused solely on the Rel homology DNA-binding domain, and obtained conflicting results that cast doubt on the domain’s true taxonomic distribution (de Mendoza et al. 2013; Richter et al. 2018; de Mendoza and Sebé-Pedrós 2019). As a result, the precise origin, early evolution, and molecular context of Rel homology domain-containing transcription factors remained unknown.

To resolve these questions, we performed a taxonomically broad survey of genomic and transcriptomic sequence data representing all major eukaryotic supergroups, including newly sequenced protistan lineages within Holozoa (Grau-Bové et al. 2017; Hehenberger et al. 2017; Tikhonenkov et al. 2020; Urrutia et al. 2021). We surveyed data from 180 species for key Rel homology DNA-binding and dimerization domain-containing proteins, examined their domain architecture, and constructed phylogenies of the proteins identified. We additionally extended the search to homologs of IKK components.

We identified RHRs in all but one of the holozoan groups examined, including the filastereans *Pigoraptor* spp., the recently sequenced *Txikispora philomaios* and *Tunicaraptor unikontum*, and several ichthyosporean species (fig. 1*B*-2; supplementary fig. S1, tables S1 and S2, Supplementary Material online). A candidate protein was also present in *Parvularia atlantis* (formerly referred to as *Nuclearia* sp. ATCC 50694 [López‐Escardó et al. 2018]), a member of the sister group to Fungi (Nucleariida), suggesting that Rel homology proteins were present in the last common ancestor of Opisthokonta, and were secondarily lost in Fungi (de Mendoza et al. 2013; de Mendoza and Sebé-Pedrós 2019). Strikingly, at least one sequence from each of these lineages included both DNA-binding and dimerization domains (fig. 1*B*-3; supplementary fig. S1 and table S2, Supplementary Material online), and C-terminal ankyrin repeat-rich regions preceded by a GRR were found in at least one sequence from Filasterea, from Ichthyosporea, and from *P. atlantis* (fig. 2; supplementary table S2, Supplementary Material online). The finding of these traits in the nucleariid *P. atlantis*, in particular, not only confirms the origin of Rel/NF-κB proteins in the opisthokont stem lineage, but also is consistent with an ancient conserved mechanism of cytosolic sequestration for these proteins. Similarly, nonmetazoan Rel/NF-κB-like sequences from all of these groups share with their animal homologs key domains for DNA-binding specificity, including a highly conserved specific recognition loop (RL) within the RHR, as well as a dimerization domain and a highly conserved monopartite NLS (figs. 3 and 4; supplementary table S2, Supplementary Material online). In contrast, the linker region between the DNA-binding and dimerization domains (Ghosh et al. 1995; Müller 1995) appears to be animal-specific (fig. 3).

**Fig. 2.**
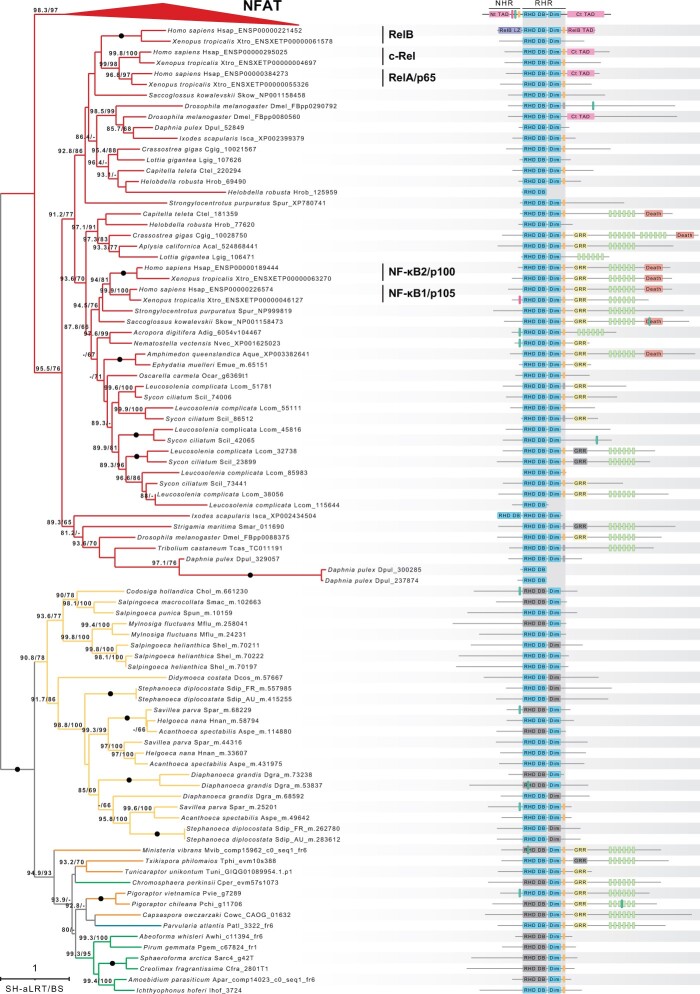
Rel homology domain phylogeny and domain architecture of Rel homology domain-containing proteins in NF-κB and NFAT families. Only SH-aLRT and nonparametric bootstrap support values above 80 and 60, respectively, are shown. Fully supported bipartitions are indicated with filled circles. Sequences are color-coded according to taxonomic group: red, Metazoa; yellow, Choanoflagellatea; orange, Filasterea; green, Ichthyosporea; and blue, Holomycota (Fungi and their closest relatives). The two side lengths of the collapsed NFAT clade are proportional to the distances between the node and its closest and furthest leaves. Schematic representation of Pfam domains and conserved regions related to Rel/NF-κB and NFAT proteins in the RHR are depicted to the right of each gene identifier; colors and abbreviations as in figure 1*A*. Domains or regions depicted in gray match domains or regions that were identified in previous Pfam versions or that share high sequence similarity in the protein alignment (at least four Lysine or Arginine residues in the case of NLS).

**Fig. 3.**
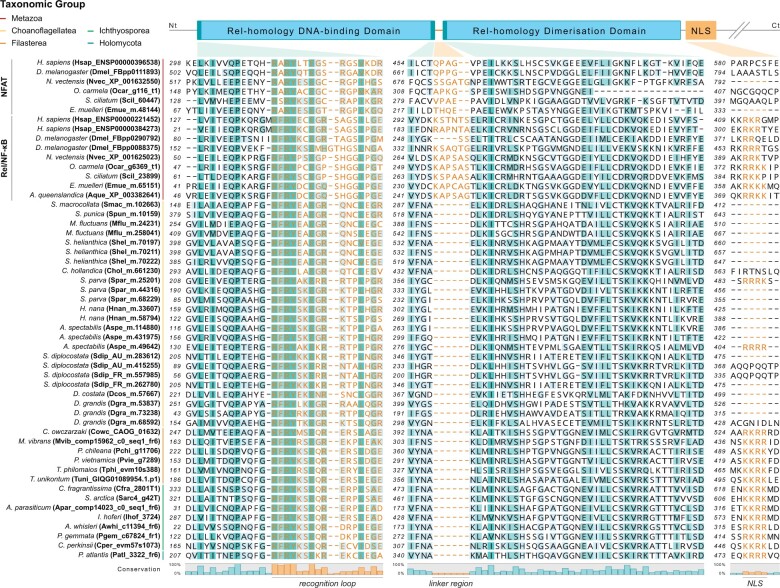
Partial multiple protein sequence alignment of key Rel/NF-κB domains in Opisthokonta. Partial protein sequence alignment (generated by MAFFT v7.299b E-INS-i multiple sequence alignment with the gap extension parameter set to 0) depicting the N- and C-terminal regions of the Rel homology DNA-binding domain, the N-terminal region of the Rel homology dimerization domain and the complete NLS. Background shading of individual amino acids reflects the degree of conservation at a given position; this degree of conservation is also depicted in the histogram shown below the alignment. Dashes indicate gaps in the alignment. Key DNA-binding amino acid residues in the recognition loop, residues comprising the complete linker region between the Rel homology DNA-binding domain and dimerization domain, and key residues in the NLS are highlighted in orange. The amino acid positions in the original sequences are shown to the left of each alignment series.

**Fig. 4.**
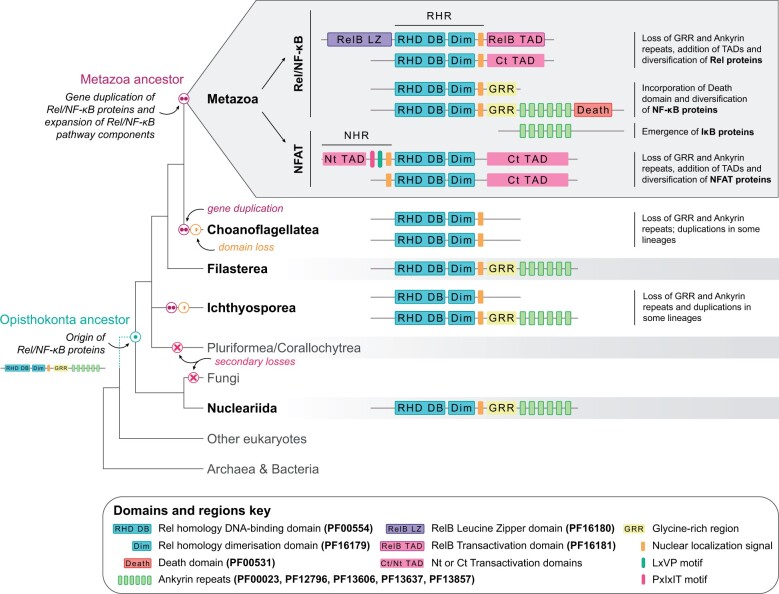
Evolution and diversification of Rel/NF-κB proteins in Opisthokonta. The Rel/NF-κB proteins originated along the Opisthokonta stem and diversified through gene duplication events and secondary losses and gains of key domains. Based on low sequence conservation of some regions and the lack of experimental information, we cannot rule out the possibility of TADs being present in other nonmetazoan species.

Metazoan Rel/NF-κB proteins and metazoan NFAT proteins each formed a well-supported clade, within a larger well-supported clade of metazoan sequences. Sequences from choanoflagellates, filastereans, and ichthyosporeans formed a clade sister to all metazoan sequences (fig. 2). The most parsimonious explanation suggested by the phylogeny is a duplication of ancestral Rel/NF-κB proteins in the metazoan stem lineage followed by loss of the GRR and ankyrin repeats from NFAT proteins (fig. 4; Sebé-Pedrós et al. [2011] and Gilmore and Wolenski [2012]), and additional duplications in individual metazoan and choanoflagellate lineages (Williams and Gilmore 2020). None of the nonmetazoan Rel/NF-κB sequences contained death domains. The animal NFAT sequences recovered in our survey contain the calcineurin-binding motifs LxVP, and, in the case of chordates, PxIxIT (Wigington et al. 2020) near the N-terminus. Seven of the nonmetazoan Rel/NF-κB-like proteins contained a LxVP motif. In the choanoflagellates *Codosiga hollandica* and *Savillea parva* (two out of three proteins), and in the filasterean *Pigoraptor vietnamica*, this motif was found near the N-terminus, before the RHR (fig. 2; supplementary table S2, Supplementary Material online). This raises the possibility that calcineurin may be an additional regulator of some nonmetazoan Rel/NF-κB-like proteins, as it is in NFAT proteins.

Despite the presence of C-terminal ankyrin-rich repeats preceded by a GRR in some sequences from nonmetazoan opisthokonts, and consistent with earlier reports (Williams and Gilmore 2020), we were unable to retrieve any apparent orthologs of NEMO (fig. 1*B*; supplementary fig. S1, Supplementary Material online) or the other IKK subunits (IKKα, IKKβ, and IKKε) outside Metazoa (data not shown). If the C-terminal region of nonmetazoan Rel/NF-κB-like proteins is processed, it may be phosphorylated by another kinase, or targeted for degradation by a different mechanism.

The diversity of nonmetazoan Rel/NF-κB-like proteins likely reflect the variety of lifestyles of the organisms in which they are found. Choanoflagellates are free-living, mostly marine or freshwater bacterivores, some of which form clonal multicellular structures in response to specific bacterial molecules (Alegado et al. 2012; Leadbeater 2015); filastereans include both free-living freshwater bacterivores and endobiotic species (Stibbs et al. 1979; Tong 1997; Hehenberger et al. 2017; Tikhonenkov et al. 2020; Urrutia et al. 2021), at least some of which can form multicellular aggregates (Sebé-Pedrós et al. 2013; Hehenberger et al. 2017; Mylnikov et al. 2019); ichthyosporeans include free-living species and parasites of invertebrates or fish, with diverse life cycles and cell states including multinucleate coenocytic stages (reviewed in Mendoza et al. [2002]); and *Parvularia* is a free-living freshwater bacterivorous amoeboid (López‐Escardó et al. 2018). Rel/NF-κB-like proteins may play similar or very different roles in how these organisms interact with a variety of prey or host organisms, and/or environmental factors. Interactions with newly evolved partners and gene duplications may have been key to increasing their combinatorial regulatory capabilities in different lineages, including along the animal stem.

Overall, we provide an updated evolutionary reconstruction of Rel/NF-κB and NFAT transcription factor families, based on a broad taxon sampling including representatives of all major eukaryotic lineages. We show that Rel/NF-κB-like proteins emerged earlier than previously known, prior to the split between animals and fungi. We further highlight conserved, animal-like architecture in these proteins from diverse opisthokonts. Together, our results suggest that localization and regulatory mechanisms found in animal Rel/NF-κB proteins were likewise present in the last common ancestor of animals and fungi.

## Materials and Methods

Raw Hidden Markov Models (HMMs) of Rel homology DNA-binding domain (RHD_DNA_bind v.21, PF00554), Rel homology Dimerization domain (RHD_dimer v.4, PF16179), Death domain (Death v.21, PF00531), and the IKK component domains Inhibitor of Kappa B Kinase Beta NEMO-binding domain (IKKbetaNEMObind v.7, PF12179) and NF-Kappa B Essential Modulator (NEMO v.7, PF11577) were retrieved from Pfam v.34.0 (Mistry et al. 2021), and used as queries in hmmscan (hmmer 3.1b2-2; Eddy 1998; Söding 2005) searches against a paneukaryotic predicted proteome database enriched in holozoan representatives (supplementary table S1, Supplementary Material online). BLAST searches for IKK complex components were carried out using *Homo sapiens* (GenBank accession numbers O15111.2 [Inhibitor of nuclear factor kappa-B kinase subunit alpha], O14920.1 [Inhibitor of nuclear factor kappa-B kinase subunit beta], Q9Y6K9.2 [NF-kappa-B essential modulator], Q14164.1 [Inhibitor of nuclear factor kappa-B kinase subunit epsilon]) and *Nematostella vectensis* (ADQ57374.1 [single IKK-like protein]) IKK complex components as queries.

Using custom Perl scripts, the resulting output files were parsed and reanalyzed using PfamScan v.1.5 (Gish and States 1993), and all sequences containing a Rel homology DNA-binding domain were retrieved and examined using reciprocal best BlastP searches against the nonredundant protein database (nr) of the National Center for Biotechnology Information (NCBI). The domain architecture of all retrieved sequences was inferred with PfamScan using the gathering threshold as cutoff value. The number of ankyrin repeats was verified using InterProScan 5.26-65.0 (Jones et al. 2014).

Sequences were aligned using MAFFT v7.299b E-INS-i (Katoh et al. 2002, 2005; Katoh and Standley 2013) with the gap extension parameter set to 0, trimmed using BMGE v.1.0 (Criscuolo and Gribaldo 2010) using the BLOSUM45 matrix, and alignments and trimming were verified by eye. Partial sequences with fewer than 50% of positions represented in the final trimmed alignment were excluded. Preliminary phylogenies were constructed using FastTree v. 2.1.9 (Price et al. 2009, 2010); the number of metazoan representatives was reduced. Final alignments were constructed using MAFFT E-INS-i with the gap extension parameter set to 0, and trimmed using trimAl v1.4.rev22 build[2015-05-21] (Capella-Gutiérrez et al. 2009), and final phylogenies were constructed using IQ-TREE multicore version 2.0-rc1 (Nguyen et al. 2015; Minh et al. 2020) with 1,000 ultrafast bootstrap resamplings (Minh et al. 2013; Hoang et al. 2018), using LG + F + R7 for Rel homology domain proteins (chosen by ModelFinder [Kalyaanamoorthy et al. 2017) as the best fitting model according to the Bayesian Information Criterion). Subsequently, 100 nonparametric bootstrap replicates were also performed under the same model.

## Supplementary Material

Supplementary data are available at *Genome Biology and Evolution* online.

## Supplementary Material

evab289_Supplementary_Data
