# Supplementary material for: Rel/NF-κB Transcription Factors Emerged at the Onset of Opisthokonts
Source: Genome Biol Evol. 2022 Jan 6;14(1):evab289. doi: 10.1093/gbe/evab289 (PMC8763368; doi:10.1093/gbe/evab289)
Supplement: evab289_Supplementary_Data [file evab289_supplementary_data.zip › Leger supplementary materials.docx]

**Supplementary materials**

**Figure S1. Number of domains related to Rel/NF-κB families and related partners in Opisthokonta (related to Figure 1).** Number of key Pfam domains analyzed in this study are represented in columns and color-coded according to presence in or absence from genome or transcriptome data source (indicated in the upper right legend).

**Table S1.** Taxon sampling and data sources used in this study, highlighting species in a which a Rel homology domain had previously been reported in specific studies.

**Table S2.** Rel homology domain-containing proteins identified in the taxa surveyed in this study, showing key domains and motifs, used as the basis for Figure 2.

**Dataset S1.** Supplementary data relating to Figure 2: Untrimmed alignment, trimmed alignment and single-protein phylogenies in Newick format
