# Supplementary material for: Rel/NF-κB Transcription Factors Emerged at the Onset of Opisthokonts
Source: Genome Biol Evol. 2022 Jan 6;14(1):evab289. doi: 10.1093/gbe/evab289 (PMC8763368; doi:10.1093/gbe/evab289)

Figure S1. Number of domains related to Rel/NF-κB families and related partners in Opisthokonta and Amoebozoa (related to Figure 1).
Number of key Pfam domains analyzed in this study are represented in columns and color-coded according to presence in or absence from genome or transcriptome data source (indicated in the upper right legend). Information on taxon sampling and Rel proteins in Table S1.

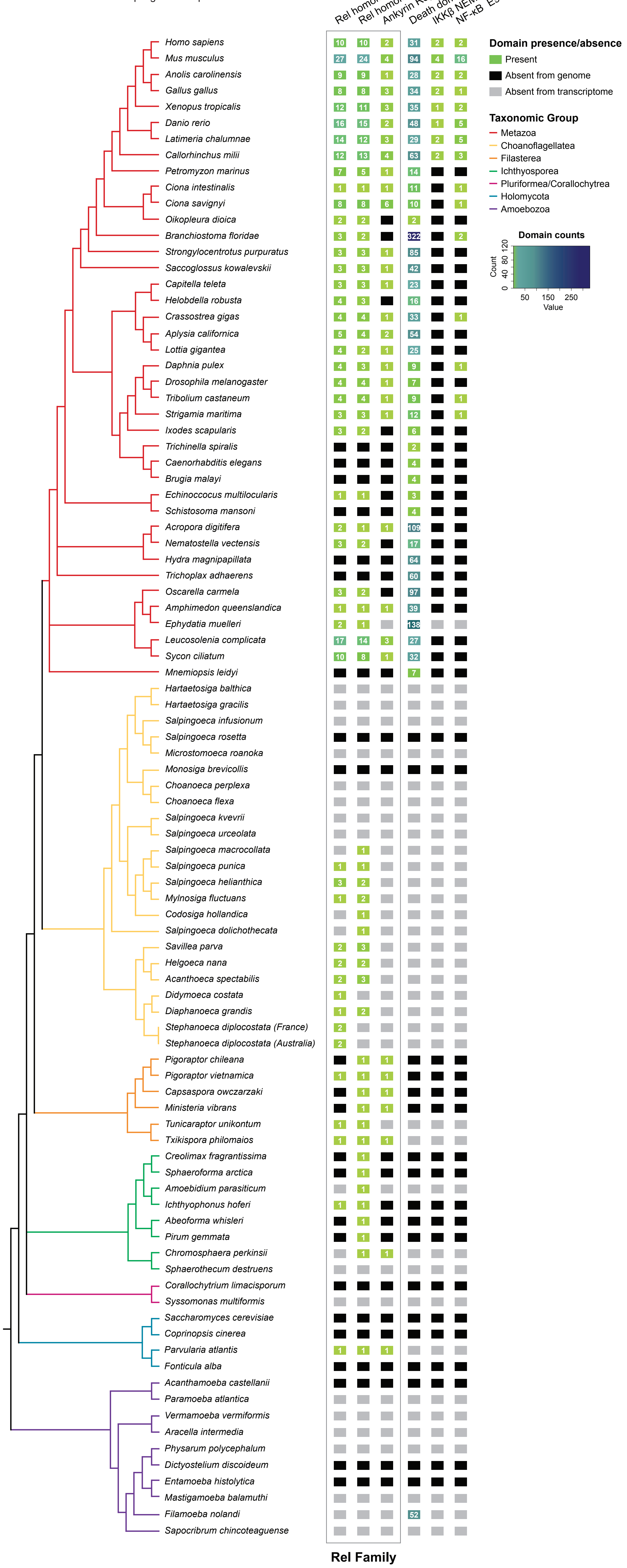

Supplement: evab289_Supplementary_Data [file evab289_supplementary_data.zip › Leger_GBE_Fig_S1rev.pdf]
